# Neuroanatomical correlates of stroke-related dizziness and vertigo: secondary analysis from the INSPiRE-TMS trial

**DOI:** 10.1007/s00415-026-14033-3

**Published:** 2026-08-12

**Authors:** Yunyou Tang, Uchralt Temuulen, Ana Sofia Rios, Ahmed Khalil, Michael Ahmadi, Andreas Horn, Matthias Endres, Heinrich J. Audebert, Anna Kufner

**Affiliations:** 1 Klinik Für Neurologie Mit Experimenteller Neurologie, Charité –Universitätsmedizin Berlin, Freie Universität Berlin and Humboldt-Universität Zu Berlin, Campus Charité Mitte, Charité Platz 1, 10117 Berlin, Germany; 2 Center for Stroke Research Berlin (CSB), Charité - Universitätsmedizin Berlin, Freie Universität Berlin and Humboldt-Universität Zu Berlin, 12203 Berlin, Germany; 3 Berlin Institute of Health, Charité – Universitätsmedizin Berlin, 10117 Berlin, Germany; 4 Institute for Network Stimulation, Department of Stereotactic and Functional Neurosurgery, University Hospital Cologne, Cologne, Germany; 5 German Center for Cardiovascular Research (Deutsches Zentrum Für Herz Kreislauferkrankungen, DZHK), Partner Site Berlin, 10117 Berlin, Germany; 6 German Center for Mental Health (Deutsches Zentrum Für Neurodegenerative Erkrankungen, Psychische Gesundheit DZPG), Partner Site Berlin, 10117 Berlin, Germany; 7 German Center for Neurodegenerative Diseases (Deutsches Zentrum Für Neurodegenerative Erkrankungen, DZNE), Partner Site Berlin, 10117 Berlin, Germany; 8 Institute of Neuroradiology, Charité –Universitätsmedizin Berlin, Berlin, Germany

**Keywords:** Ischemic stroke, Magnetic Resonance Imaging, Dizziness, Vertigo, Diagnostic imaging

## Abstract

Dizziness and vertigo are common and debilitating neurological symptoms among patients visiting the emergency department (ED). Possibly because the symptoms are not captured by standard stroke severity tools such as the NIHSS (National Institutes of Health Stroke Scale), their network-level neuroanatomical correlates—particularly potential supratentorial contributions—remain incompletely characterized. Therefore, we aim to investigate the neuroanatomical substrates of post-stroke dizziness and vertigo. This retrospective analysis included patients with acute ischemic stroke from the randomized controlled INSPiRE-TMS trial. Each patient underwent MRI within seven days of symptom onset, and stroke severity was assessed using the NIHSS at hospital admission. Patients were assessed for broadly defined new-onset dizziness/vertigo associated with the acute cerebrovascular event. Lesion symptom mapping (LSM) and lesion network mapping (LNM) were used to investigate associations between lesion location, functional connectivity, and dizziness/vertigo. Among 484 patients, 32% (*n* = 157) reported dizziness at stroke onset. Notably, dizziness was reported in 22% (77/347) of patients with supratentorial lesions, compared with 58% (80/137) of those with infratentorial lesions. Patients with dizziness were more often female (36.9% vs. 27.8%, *p* = 0.042) and more frequently had hemianopia (35.1% vs. 15.6%, *p* < 0.0001). LSM identified cerebellar clusters (left lobules VI, Crus I–II, VIIb, VIIIa, and bilateral lobule IX with the vermis) associated with visuospatial and multisensory processing. LNM revealed a dizziness network involving left-dominant cerebellar regions, the brainstem, and higher-order visual areas, including the occipital pole, lingual gyrus, and fusiform gyrus. This network remained robust across subgroups stratified by lesion location (supra- vs. infratentorial) and after adjusting for age, sex, lesion volume, and hemianopia. Exploratory subtype comparisons suggested that lesions associated with vertigo showed relatively stronger connectivity to midline cerebellar structures (vermis), whereas those associated with swaying dizziness involved broader cerebellar hemispheres, although the overall network pattern was spatially similar and centered on the cerebellum. In conclusion, broadly defined post-stroke dizziness/vertigo symptoms were associated with a consistent lesion network involving cerebellar and occipital regions, suggesting involvement of distributed visual, vestibular, and multisensory brain systems. These findings may help guide future studies aimed at improving diagnostic evaluation and treatment of affected patients. ClinicalTrials.gov (NCT01586702; https://clinicaltrials.gov/ct2/show/NCT01586702); first registry in April 2012; first patient recruitment: September 2011.

**Supplementary Information:**

The online version contains supplementary material available at https://doi.org/10.1007/s00415-026-14033-3.

## Introduction

Dizziness/vertigo are common symptoms and account for up to 4% of all emergency department (ED) visits [1]. For this manuscript, we will subsume the two with “dizziness” unless specifically segregated into vertigo and swaying dizziness. Evaluating patients who present with dizziness as their primary complaint poses a diagnostic challenge, given that its causes range from benign conditions such as vestibular migraine to high-risk etiologies like ischemic stroke in the vertebrobasilar (VB) circulation [2]. Stroke-related dizziness accounts for up to 5% of dizziness presentations in the ED [1], where timely and accurate diagnosis is critical for initiating appropriate acute treatment.

Dizziness is a debilitating symptom of ischemic stroke that is not captured by standard stroke severity scales, such as the NIHSS, and is therefore often understudied [3]. Understanding the neuroanatomical correlates of post-stroke dizziness may facilitate improved diagnostic evaluation and inform future therapeutic strategies.

Dizziness is more common in older adults and females [4], and may be caused by VB strokes that affect vestibular pathways in the brainstem and cerebellum [5, 6]. However, cortical lesions can also cause dizziness [7–9], though isolated vestibular symptoms from supratentorial strokes are rare (~ 4%) [10]. The vestibular cortex––centered around parietal operculum area 2 (OP2)––plays a key role in multisensory integration [11, 12]. Recent lesion network mapping (LNM) studies highlight cortical networks involving OP2 and the posterior insula in cortical vertigo [13], although unilateral cortical lesions alone may not suffice to cause vertigo, likely due to interhemispheric compensation [14, 15]. However, prior network-level analyses suggest that functional connectivity mapping alone may be insufficient to distinguish vestibular cortical lesions that elicit vertigo from those that do not [16].

These findings underscore the complexity of stroke-related dizziness and the need to better understand potential supratentorial mechanisms. To address this, we investigated the neuroanatomical correlates of dizziness in a large, prospective cohort from the multicenter randomized INSPiRE-TMS trial. Our objectives were to (1) identify clinical parameters associated with dizziness; (2) localize lesion sites associated with dizziness using lesion symptom mapping (LSM); and (3) characterize brain networks associated with dizziness, including potential supratentorial contributions. For the latter, we stratified analyses by (3a) lesion location (supratentorial vs. infratentorial) and (3b) dizziness phenotype (vertigo vs. swaying dizziness).

## Materials and methods

### Participants

This study is a retrospective analysis of patient data derived from the INSPiRE-TMS trial (Intensified Secondary Prevention intending a Reduction of vascular re-events after TIA or Minor Stroke; ClinicalTrials.gov: NCT01586702). In brief, INSPiRE-TMS was a multicenter, randomized controlled trial designed to evaluate the impact of an intensified secondary prevention program in patients with minor ischemic stroke (modified Rankin Scale ≤ 2) or transient ischemic attack (TIA) (ABCD_2_ score ≥ 3). For a detailed description of the main trial data, and inclusion and exclusion criteria, refer to INSPiRE-TMS program description [17].

For the current study, patients were included if they met the following criteria: (1) availability of MRI acquired as part of the routine clinical diagnostic workup during the acute hospital stay; (2) presence of a visible ischemic lesion on MRI within seven days of symptom onset; (3) clinical assessment by the attending physician upon admission to the stroke unit; and (4) available documentation of the assessment of the symptom dizziness at the time of admission. Exclusion criteria was the failure to normalize lesions to a common space due to poor image quality.

The trial was approved by the Ethics Committee of Charité Universitätsmedizin Berlin (EA2/084/11) and received additional approval from the respective ethics committees at all participating centers.

### Baseline assessment

All patients underwent a neurological examination upon admission to the ED and stroke severity was evaluated via the NIHSS by the attending physician. Trained study personnel prospectively questioned patients upon admission to the stroke unit for the presence of predefined symptoms related to the acute cerebrovascular event, including new-onset dizziness (yes/no/not specified) [18]. Of note, according to the International Classification of vestibular symptoms, “dizziness” and “vertigo” are defined as different types of vestibular symptoms: “dizziness” refers to a disturbed or impaired sense of spatial orientation without a false sensation of motion, while “vertigo” specifically involves the false perception of motion [19]. However, in the German language the term “*Schwindel*” is commonly used as an umbrella term for both entities [20]. In this study, "dizziness" therefore is translated from the German "*Schwindel,*" so our analyses address broadly defined post-stroke dizziness rather than a standardized vestibular diagnosis. Further, reported “*Schwindel*” was retrospectively categorized into vertigo vs. swaying dizziness in a post hoc analysis based on ED records described below.

During the patients’ stay on the stroke unit, study personnel documented demographic information and cerebrovascular risk factors—including history of hypertension, diabetes, hyperlipidemia, atrial fibrillation, smoking, and previous stroke. Stroke etiology was determined and classified according to the Trial of Org 10,172 in Acute Stroke Treatment (TOAST) classification criteria [21]. Within seven days of the index event (during the acute hospital stay), patients completed the EQ‑5D‑5L, a standardized, patient-reported instrument used to assess health-related quality of life. The EQ‑5D‑5L evaluates the following five dimensions: mobility, self-care, usual activities, pain/discomfort, and anxiety/depression [22].

### Neuroimaging

As is typical in clinical samples, neuroimaging was acquired using different scanners across sites. All patients underwent MRI during their acute hospital stay (within 7 days of stroke onset) and received a standardized stroke imaging protocol performed on either a 1.5-Tesla (n = 56) or 3-Tesla scanner (n = 428). Sequences included a T2*-weighted sequence, diffusion-weighted imaging (DWI), "time-of-flight" MR angiography, and a fluid-attenuated inversion recovery (FLAIR) sequence. The DWI protocol consisted of images acquired with a b-value of 1000 s/mm^2^, non-diffusion-weighted images (b = 0 s/mm^2^), and an apparent diffusion coefficient map.

#### MRI data preprocessing

**Fig. 1:**
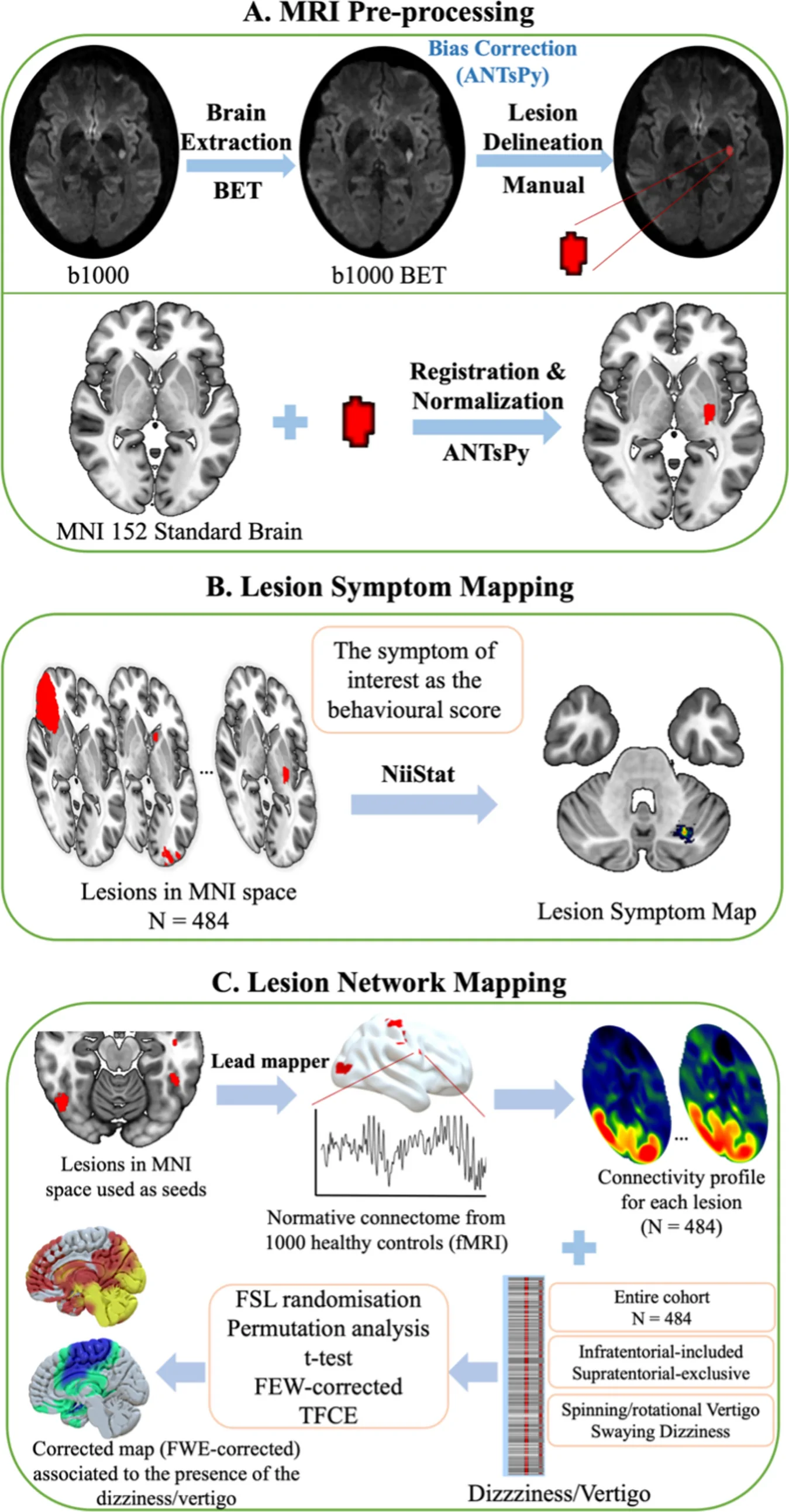
Workflow of MRI preprocessing, lesion symptom and network mapping. **A** MRI preprocessing pipeline. Brain extraction was performed on b1000 images using BET. Bias correction was applied using ANTsPy. Lesions were manually delineated. Brain-extracted images and lesion masks were co-registered and normalized directly to the MNI152 standard space. **B** Lesion symptom mapping workflow. Normalized lesion maps and dizziness status were entered into NiiStat. Voxels lesioned in at least 5% of dizzy patients were included in the analysis according to previously published studies [30]. **C** Lesion network mapping workflow. Lesion masks were used as seeds in Lead Mapper to compute voxel-wise functional connectivity using a normative connectome derived from resting-state fMRI data of 1000 healthy controls [36, 37]. Connectivity profiles were concatenated and entered a permutation-based two-sample t-test to compare patients with and without dizziness (5000 permutations per contrast). Resulting maps were family-wise error (FWE) corrected, and threshold-free cluster enhancement (TFCE) was applied

DWI images were skull stripped using the Brain Extraction Tool (BET) from the FMRIB Software Library (FSL) [23–25]. Ischemic lesions were manually delineated on DWI and FLAIR sequences using MRIcron [26] by an experienced physician (A.S.R) blinded to clinical data and supervised by at least two independent expert neurologists and/or radiologists (A.Ku, A.Kh). After bias correction to remove the intensity inhomogeneity artefacts with Advanced Normalization Tools in Python (ANTsPy), the resulting lesion masks were co-registered and normalized into the Montreal Neurological Institute (MNI) common space (MNI152 atlas, 1 × 1 × 1 mm) by performing a series of linear and non-linear registrations following lesion masking with ANTsPy [27]. For a graphical depiction of MRI preprocessing steps and subsequent imaging analyses (including lesion symptom and lesion network mapping), refer to Fig. 1.

The affected arterial territories of the lesions were quantified by superimposing the Digital 3D Brain MRI Arterial Territories Atlas onto the lesions in MNI space [28]. Specifically, lesions within the middle cerebral artery (MCA) territory involved the lateral lenticulostriate territory, the "lobar" MCA territory (cortex and adjacent subcortical white matter), or both. Lesions within the posterior cerebral artery (PCA) affected the posterior and anterior thalamic-perforating territories, the "lobar" PCA territory, or a combination of these. Similarly, lesions within the anterior cerebral artery (ACA) infarcts affected the medial lenticulostriate territory, the "lobar" ACA territory, or both. Vertebrobasilar (VB) infarcts were localized to the inferior or superior cerebellar areas, the basilar territory, or a combination. Infarcts spanning multiple arterial territories affected combinations of the regions mentioned above.

#### Lesion symptom mapping (LSM)

We performed voxel-wise and atlas-based LSM to explore the association between lesion location and the presence of dizziness (binary variable) following ischemic stroke using the NiiStat Software [29]. Non-parametric Liebermeister measures were used for binary symptom analysis. Lesions of a total of 484 patients were included in the analysis; only voxels lesioned in at least 8 patients (5% of patients with dizziness) were considered [30]. At each voxel, a one-tailed t-test was performed to test the association between lesions and the presence of dizziness. Statistical significance was determined based on normal permutation-derived z-score thresholds (n = 5000), where p was < 0.05. Lesion volume was included as a covariate in the model. Atlas-based LSM results were labeled using the JHU atlas, which includes 189 cortical and subcortical regions [31]. Significantly associated clusters identified in voxel-based LSM were anatomically labeled using the Harvard–Oxford Cortical and Subcortical Structural Atlases and the FNIRT-normalized Cerebellar Atlas in MNI152 space [32, 33]. The cerebellar flatmap with significant voxels was generated using the SUIT toolbox [34].

#### Lesion network mapping (LNM)

LNM was conducted following established methodologies described in detail previously [35]. Briefly, normalized binary lesion masks from individual participants were used as seeds to estimate functional connectivity based on a normative functional connectome derived from resting-state fMRI data of 1,000 healthy individuals [36, 37] using the Lead Mapper from Lead-DBS (lead-dbs.org) [38]. For each subject, voxel-wise connectivity maps were generated by correlating the average Blood Oxygen Level-Dependent (BOLD) signal within the lesion mask to every other voxel in the normative dataset. This process yields a unique connectivity profile for each lesion mask, comprising Pearson correlation coefficients, which were then Fisher z transformed.

To identify brain regions connected to stroke lesions in patients who experienced dizziness, we performed a permutation analysis using individual connectivity maps as the dependent variable and the presence of dizziness (binary variable) as the independent variable using the FSL tool, randomize [23, 39] with a general linear model (GLM) design equivalent to a two-sample *t*-test. Two contrasts were defined: [1, −1] to identify regions functionally connected to lesions associated with the presence of dizziness, and [−1, 1] to identify regions connected to lesions in patients without dizziness. Each contrast was tested using 5,000 permutations. Results were corrected for multiple comparisons using family-wise error (FWE) correction, and a threshold-free cluster enhancement (TFCE) was applied to detect cluster-forming voxels within connected regions. [40] These maps represent 1 minus the P value of the statistical association between dizziness and the normative connectivity profile. Regions were considered significantly connected to lesion sites if the corrected value exceeded 0.95 (i.e., pFWE < 0.05). Significant voxels were overlaid onto standard structural atlases to localize the connected brain regions, including the Harvard–Oxford Cortical (HOOC) and Subcortical Structural Atlases (HOOS) [32], as well as the FNIRT-normalized Cerebellar Atlas in MNI152 space [33]. This analysis was conducted in three steps: (1) across the entire cohort, irrespective of lesion location; (2) within two subcohorts stratified by lesion location (supratentorial vs. infratentorial); and (3) according to dizziness subtype (spinning/rotational vertigo vs. swaying dizziness).

#### Sensitivity analysis

Two sensitivity analyses were conducted to assess the robustness of the dizziness-related network derived from LNM. First, an adjusted LNM was performed by incorporating the following covariates into the model: age, sex, lesion volume, and the presence of hemianopia in the GLM model. Age and sex were included as established demographic predictors of dizziness [4], while lesion volume was included to control for potential effects of lesion size on connectivity [30]. Hemianopia was included due to its significantly higher prevalence among patients reporting dizziness (Table 1). We note, however, that hemianopia and dizziness may both arise from shared PCA territory lesions, so this adjustment was intended as an exploratory robustness check rather than as a definitive confounding control.

**Table 1:** Patient demographics and clinical characteristics of the entire cohort and based on the presence/absence of dizziness upon admission. Statistical comparisons in Table 1 are provided for descriptive purposes and were not interpreted as confirmatory evidence; therefore, no correction for multiple comparisons was applied

	Total	Dizziness	No Dizziness	*P* value
	*N* = 484	*N* = 157	*N* = 327	
Age, mean (± SD)	67.3 (10.7)	66.7(10.8)	67.6 (10.6)	0.526
Sex, female, n (%)	149 (30.8)	58 (36.9)	91 (27.8)	0.042*
Hypertension, n (%)	407 (84.1)	133 (84.7)	274 (83.8)	0.795
Diabetes, n (%)	116 (24.0)	30 (19.1)	86 (26.3)	0.083
Hyperlipidemia, n (%)	136 (29.1)	51 (34.0)	85 (26.7)	0.488
Atrial fibrillation, n (%)	84 (17.4)	26 (16.6)	58 (17.7)	0.749
History of smoking, n (%)	313 (64.7)	96 (61.2)	217 (66.4)	0.49
History of stroke, n (%)	90 (19.0)	27 (17.8)	63 (19.6)	0.641
TOAST Classification [21]				0.193
Large artery atherosclerosis, n (%)	80 (16.6)	29 (18.7)	51 (15.6)	
Cardioembolic stroke, n (%)	80 (16.6)	24 (15.5)	56 (17.1)	
Small vessel occlusion, n (%)	73 (15.2)	18 (11.6)	55 (16.8)	
Other, n (%)	8 (1.7)	5 (3.2)	3 (0.9)	
Unknown, n (%)	241 (50.0)	79 (51.0)	162 (49.5)	
ARWMC score, median (IQR)	6 (4–9)	5 (3–8)	6 (4–9)	0.184
DWI lesion volume at baseline (ml), median (IQR)	0.96 (0.35–3.55)	1.01 (0.34–5.11)	0.94 (0.36–2.92)	0.5157
NIHSS at admission, median (IQR)	2 (1–3)	2 (1–3)	2 (1–3)	0.545
Arterial territory [28]				< 0.0001*
Middle Cerebral Artery (MCA), n (%)	41 (8.5)	3 (1.9)	38 (11.6)	
Anterior Cerebral Artery (ACA), n (%)	4 (1.0)	2 (1.3)	2 (0.6)	
Posterior Cerebral Artery (PCA), n (%)	21 (4.3)	6 (3.8)	15 (4.6)	
Vertebrobasilar arteries (VB), n (%)	39 (8.1)	25 (15.9)	14 (4.3)	
Multiple arterial territories, n (%)	379 (78.3)	123 (78.3)	256 (78.3)	
Lesion location				< 0.0001*
Supratentorial lesions, n (%)	347 (71.7)	77 (49.0)	270 (82.6)	
Any Infratentorial lesions, n (%)	137 (28.3)	80 (51.0)	57 (17.4)	
Baseline EQ-5D-5L				
Anxiety/depression, n (%)	116 (26.5)	40 (27.8)	76 (25.9)	0.682
Impaired Mobility, n (%),	129 (29.5)	53 (36.8)	76 (25.9)	0.019*
Pain/discomfort, n (%)	182 (41.7)	57 (39.6)	125 (42.7)	0.539
Problems in self-care, n (%)	32 (7.3)	11 (7.6)	21 (7.1)	0.859
Problems in usual activity, n (%)	85 (19.45)	33 (22.92)	52 (17.75)	0.199
Accompanying neurological deficits				
Disorientation, n (%)	0 (0)	0 (0)	0 (0)	1
Paresis, n (%)	255 (53.8)	64 (41.0)	191 (60.1)	0.0001*
Aphasia, n (%)	85 (18.1)	12 (7.8)	73 (23.03)	< 0.0001*
Dysarthria, n (%)	165 (35.1)	59 (38.6)	106 (33.4)	0.276
Hemianopia, n (%)	104 (21.9)	54 (35.1)	50 (15.6)	< 0.0001*
Sensory deficits, n (%)	178 (37.1)	57 (37.0)	121 (37.1)	0.983

* Indicates statistical significance at the P value < 0.05 level.

Second, given prior reports suggesting that MRI preprocessing steps can substantially influence downstream imaging analyses [41], we repeated the unadjusted LNM using an alternative image preprocessing pipeline. Specifically, all lesions were re-registered to standard space using this alternative pipeline before rerunning the LNM analysis in the full cohort. The details for both the original and alternative preprocessing workflows are provided in Supplementary Table S1.

#### Infratentorial vs. supratentorial lesions associated with dizziness

To explore region-specific contributions to dizziness, patients were stratified into two groups based on lesion location: those with lesions restricted to supratentorial regions (cerebral hemispheres, diencephalon, and basal ganglia; n = 347) and those with lesions involving infratentorial regions (brainstem and cerebellum; n = 137). Lesion assignment was initially guided by the Digital 3D Brain MRI Arterial Territories Atlas, [28] followed by a visual review to further confirm group allocation. The latter group included patients with both lesions confined to the infratentorial compartment as well as those with both infra- and supratentorial lesions. This stratification was conducted to enable the identification of potentially distinct dizziness-related networks, differentiating those attributable to cortical (supratentorial) versus infratentorial lesion sites.

Here, we re-ran unadjusted LNM analyses for dizziness as described in detail above in both subgroups. For the supratentorial subgroup, we performed an adjusted LNM analysis controlling for age, sex, lesion volume, and the presence of hemianopia, and compared it to the unadjusted network as a sensitivity analysis.

#### Post hoc exploratory analysis of dizziness subtypes: vertigo vs. swaying dizziness

To further assess different dizziness phenotypes, ED medical records were retrospectively reviewed. A neurologist (A. Ku), blinded to the imaging data, evaluated the clinical notes documented by the consulting ED neurologists. Based on the terminology used during the initial clinical assessment, dizziness was categorized as either spinning/rotational vertigo (German term: “*Drehschwindel,”* hereafter referred to as vertigo) or swaying dizziness (“*Schwankschwindel”*). Cases describing only non-specific symptoms––such as light-headedness, gait instability, or drowsiness––were classified as “other.” These subtype categories represent descriptive clinical labels derived from routine ED documentation rather than standardized vestibular diagnoses, and no structured vestibular or ocular-motor assessment was performed to confirm them.

To ensure balanced group comparisons in subsequent LNM analyses, non-dizzy patients were matched with each dizziness subgroup (vertigo or swaying dizziness) in a 1:3 ratio using the MatchIt package in R [42] based on age and sex. Analogous to the LNM analyses described above, we conducted separate unadjusted analyses for the vertigo and swaying dizziness subcohorts, using individual connectivity maps as the dependent variable and dizziness subtype (binary) as the independent variable.

#### The spatial similarity analysis

Spatial similarity across networks was assessed using voxel-wise Spearman rank correlations of unthresholded, FWE-corrected network maps. Statistical significance was assessed with Moran’s spectral randomization (MSR) as implemented in the BrainSpace toolbox [43, 44]. MSR exploits the eigenvectors of a spatial weight matrix to compare the observed correlation against a null distribution of spatially autocorrelated surrogate maps. We defined the spatial weights as the inverse squared inter-voxel distance to avoid arbitrary distance thresholds and to prevent over-weighting distant voxels. For each comparison, 10,000 surrogate maps were generated, and p-values were computed as the proportion of null correlations greater than or equal to the observed correlation [45–47]. All networks were down-sampled to 4-mm isotropic resolution for this analysis only, to improve computational efficiency while preserving spatial structure.

## Results

### Cohort description

A total of 484 patients were included in the final analysis, with a mean age of 67 years (standard deviation: 10.7 years); 31% were female. The median NIHSS score on admission was 2 (interquartile range [IQR] 1–3). Patient demographics for the entire cohort, along with group comparisons based on the presence or absence of dizziness at admission, are summarized in Table 1.

Patients presenting with dizziness (*n* = 157) were more likely to be female (37%) compared to those without dizziness (*n* = 327, 28% female; *p* = 0.042). The groups did not differ significantly in age or the prevalence of common cerebrovascular risk factors (e.g., hypertension, diabetes). Additionally, no significant differences were observed in terms of stroke severity, lesion volume, stroke etiology, or age-related white matter changes (ARWMC score). Strokes involving the VB arterial territory were more common in patients with dizziness than in those without (15% vs. 4%; *p* < 0.001).

Six patients (1.2% of the entire cohort) reported dizziness as the only presenting symptom at stroke onset. In terms of accompanying neurological deficits, patients with dizziness had lower rates of accompanying paresis (41% vs. 60%, *p* < 0.001) and aphasia (8% vs. 23%, *p* < 0.001) compared to those without dizziness. In contrast, hemianopia was more frequent in patients with dizziness (35% vs. 16%, *p* < 0.001). In the self-reported QoL score EQ-5D-5L, patients with dizziness reported higher rates of subjective mobility deficits (37% vs. 26%, *p* = 0.019).

### Lesion distribution across groups

**Fig. 2:**
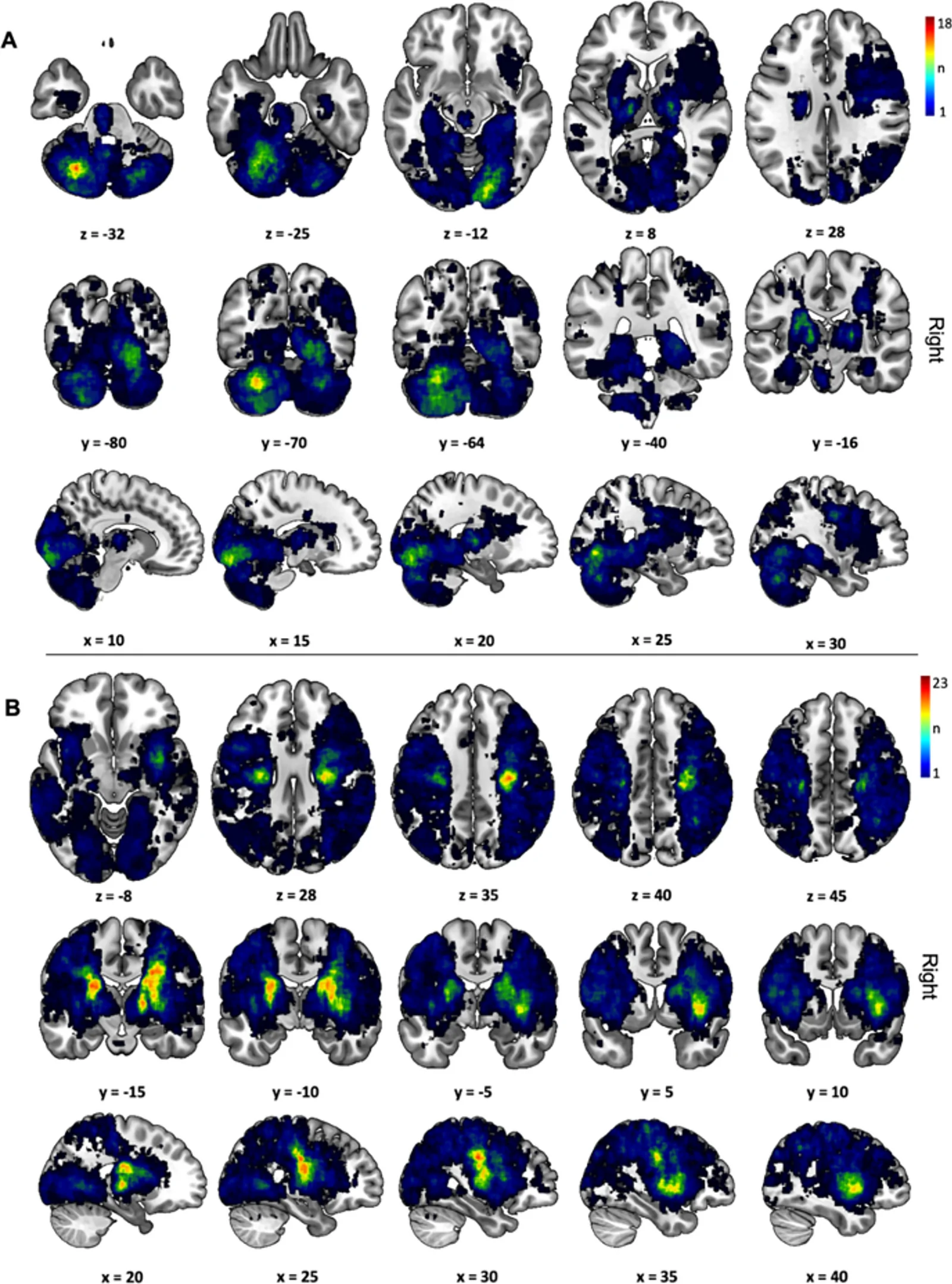
Heatmap showing lesion overlap in ischemic stroke patients with **A** dizziness (*n* = 157) and **B** without dizziness (*n* = 327) upon admission, overlaid on a 100 µm MNI brain template. The color bar reflects the voxel-wise lesion overlap, with values increasing from blue to red. Maximum lesion overlap was 18 for the dizziness group and 23 for the non-dizziness group

For the distribution of lesions included in the analysis (*n* = 484) stratified based on the presence of dizziness, refer to Fig. 2. In patients with dizziness (Fig. 2A), 49% (*n* = 77) of lesions were located exclusively in supratentorial regions, while 51% (n = 80) of lesions affected infratentorial regions. On the other hand, in non-dizzy patients (Fig. 2B), 83% (*n* = 270) of lesions were located exclusively supratentorial, while 17% (*n* = 57) also affected infratentorial regions. Regarding hemispheric lateralization, 30% (*n* = 47/157) of patients with dizziness had left-hemispheric lesions, while 27% had right-hemispheric lesions. Among non-dizzy patients (*n* = 327), 44% had lesions in the left hemisphere and 40% in the right hemisphere.

### Lesion symptom mapping (LSM)

LSM was performed in the full cohort (*N* = 484), independent of lesion location and dizziness subtype. To ensure sufficient statistical power, a minimum lesion overlap threshold of 8 patients (5% of patients with post-stroke dizziness) was applied, resulting in the inclusion of 2.2% of total voxels in voxel-based LSM. Atlas-based LSM included 153 out of 189 atlas-defined regions.

**Fig. 3:**
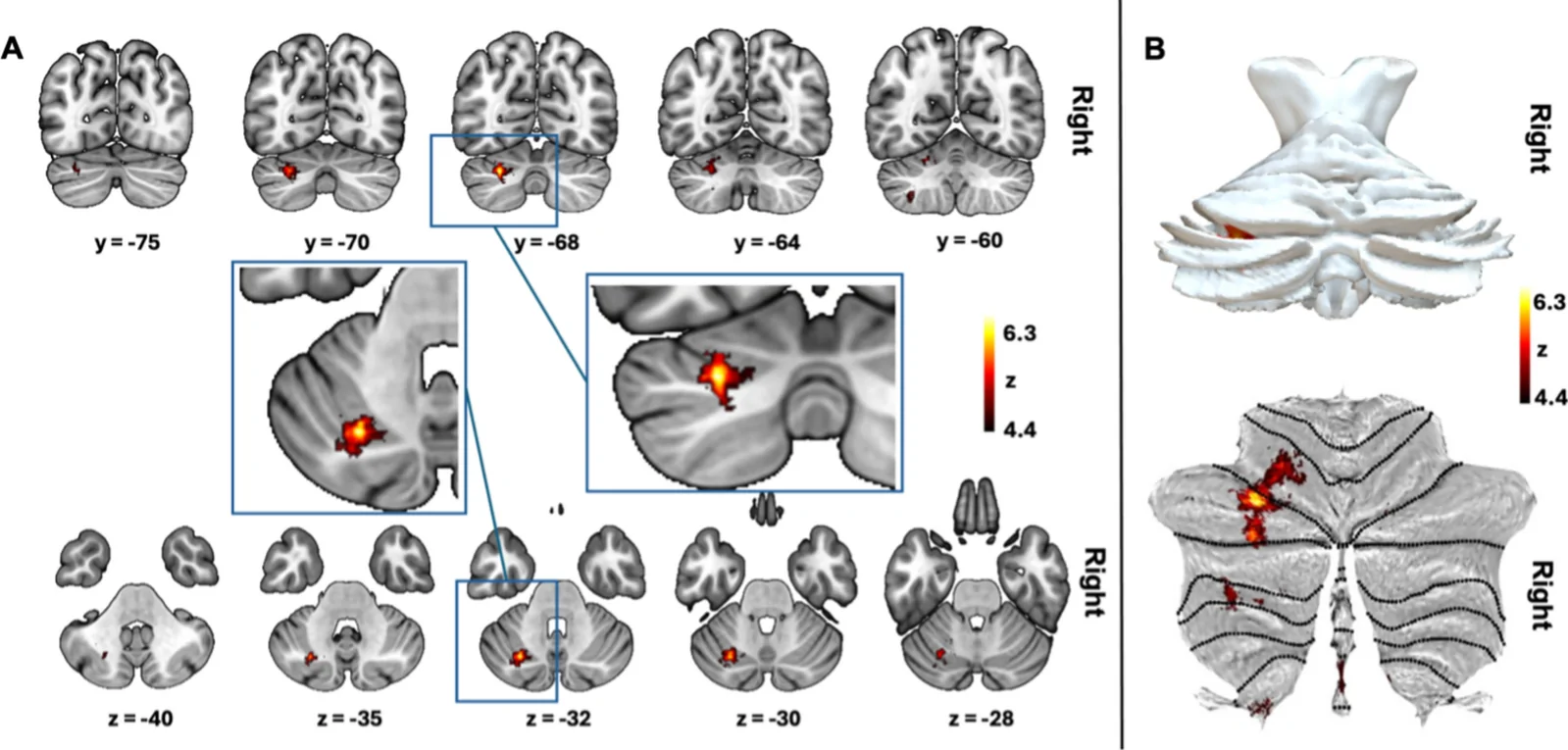
Voxel-based LSM of dizziness (binary variable) in the entire patient cohort (*N* = 484), irrespective of lesion location and dizziness subtype. **A** Voxels significantly associated with dizziness (z > 4.4) were located in the Left VI, Left Crus I, Left Crus II, Left VIIb, Left VIIIa, Left IX, Vermis IX, and Right IX cerebellar lobules. Regions with peak z-scores are shown in magnified views. **B** The 3D-rendered cerebellar surface (top) and the cerebellar flatmap (bottom) both show significant voxels. The color bars in A and B represent z-scores for the significant voxels

The permutation-derived z-score threshold corresponding to *p* < 0.05 (*n* = 5000 permutations) was z > 3.40 for the atlas-based analysis. Significant associations were found in the left cerebellum, left superior cerebellar peduncle, left middle cerebellar peduncle, left inferior cerebellar peduncle, and left pons. For voxel-based LSM, the permutation-derived significance threshold was z > 4.4 for *p* < 0.05. Significant voxels were primarily located in the cerebellum, including the Left Lobule VI, Left Crus I-II, Left VIIb, Left VIIIa, bilateral IX, and Vermis IX (Fig. 3A and B).

The significant cluster identified by LSM served as a seed region for whole-brain functional connectivity analysis, generating a connectivity profile based on Pearson correlation coefficients that were subsequently Fisher z transformed, as described above in the Methods section. The connectivity map showed positive connectivity between the LSM cluster and occipital cortical regions, including the superior and inferior divisions of lateral occipital cortex, occipital pole, occipital fusiform gyrus, and lingual gyrus.

### Functional connectivity correlates of stroke-related dizziness

**Fig. 4:**
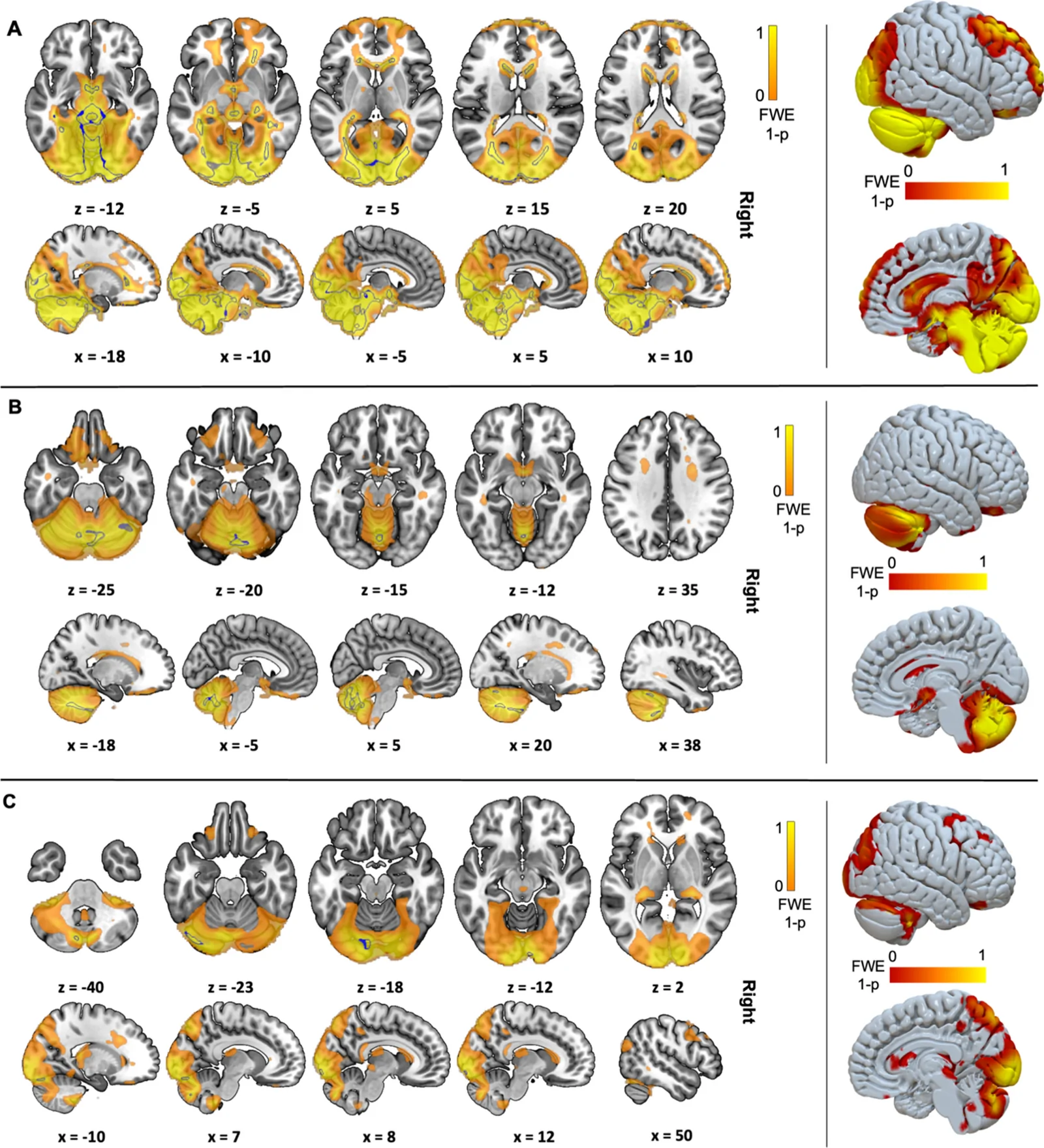
Dizziness-related symptom networks. **A** Dizziness-related network in the entire patient cohort (*N* = 484), irrespective of lesion location or dizziness subtype. **B*** Infratentorial dizziness-related network* (*n* = 137). **C*** Supratentorial dizziness-related network* (*n* = 347). The color bar illustrates the FWE 1-p values ranging from 0 to 1, with darker to lighter orange indicating increasing positive connectivity. Solid contours delineate regions of significant positive connectivity (FWE 1-p ≥ 0.95). The right panel shows the corresponding 3D renderings. The color scale transitions from red to yellow to represent increasing FWE (1-p) values, ranging from 0 to 1

The (unadjusted) LNM analysis of dizziness (binary variable: yes/no) across the entire cohort—regardless of lesion location (supratentorial or infratentorial)—revealed a largely symmetrical (bi-hemispheric) pattern of statistically significant (pFWE < 0.05) lesion connectivity, involving the cerebellum, brainstem, and occipital lobes (Fig. 4A). For a detailed list of connected regions identified through the atlas-based query, refer to Supplementary Table S2. Both sensitivity analyses—covariate-adjusted LNM for dizziness and LNM using an alternative MRI preprocessing pipeline—produced FWE-corrected spatial networks that were statistically significantly similar to the unadjusted dizziness network, as determined by MSR with 10,000 spatial surrogates (p < 0.001; Spearman r = 0.85 and 0.97, respectively). The corresponding 1-pFWE values for networks in both sensitivity analyses ranged from 0 to 1. Sensitivity analysis networks are shown in Supplementary Fig. S1; for a detailed list of connected regions, refer to Supplementary Table S3.

#### Infratentorial vs. supratentorial lesions

We repeated the same analysis as described above, but stratified lesions based on lesion location to infratentorial versus supratentorial regions of the brain. In the subgroup of patients with lesions involving infratentorial structures (*n* = 137, thereof 80 with and 57 without dizziness), LNM of dizziness showed a network centralized around the cerebellum (pFWE < 0.05) including the bilateral hemispheric and vermal portions of cerebellar lobules VI, Crus I–II, VIIb, VIIIa–b, IX, and X, hereafter referred to as the *infratentorial dizziness-related network* (Fig. 4B**)***.* LNM analysis of patients with lesions exclusively supratentorial (*n* = 77 with dizziness, *n* = 270 with no dizziness), revealed a statistically significant (pFWE < 0.05) network strongly connected to left-sided cerebellar lobules including the lobules VI, Crus I, and Crus II but also showing significant connectivity to the occipital cortex, including the inferior division of lateral occipital cortex (LOCi), the occipital pole, the lingual and fusiform gyri, hereafter referred to as the *supratentorial dizziness-related network *(Fig. 4C). For a detailed description of connected regions in each respective network, refer to Supplementary Table S2.

The infratentorial and supratentorial dizziness-related networks showed partial spatial overlap with each other (MSR; Spearman r = 0.24, *p* = 0.015), and each was statistically significantly similar to the dizziness-related network derived from the whole cohort (r = 0.69, *p* < 0.001 for the infratentorial network; Spearman r = 0.40, *p* < 0.001 for the supratentorial network).

Of note, the adjusted *supratentorial dizziness-related network* (*n* = 331), controlling for age, sex, lesion volume, and the presence of hemianopia, closely matched the unadjusted network (*n* = 347; Spearman* r* = 0.82, *p* < 0.001; Supplementary Fig. S1C).

#### Vertigo vs. swaying dizziness

**Fig. 5:**
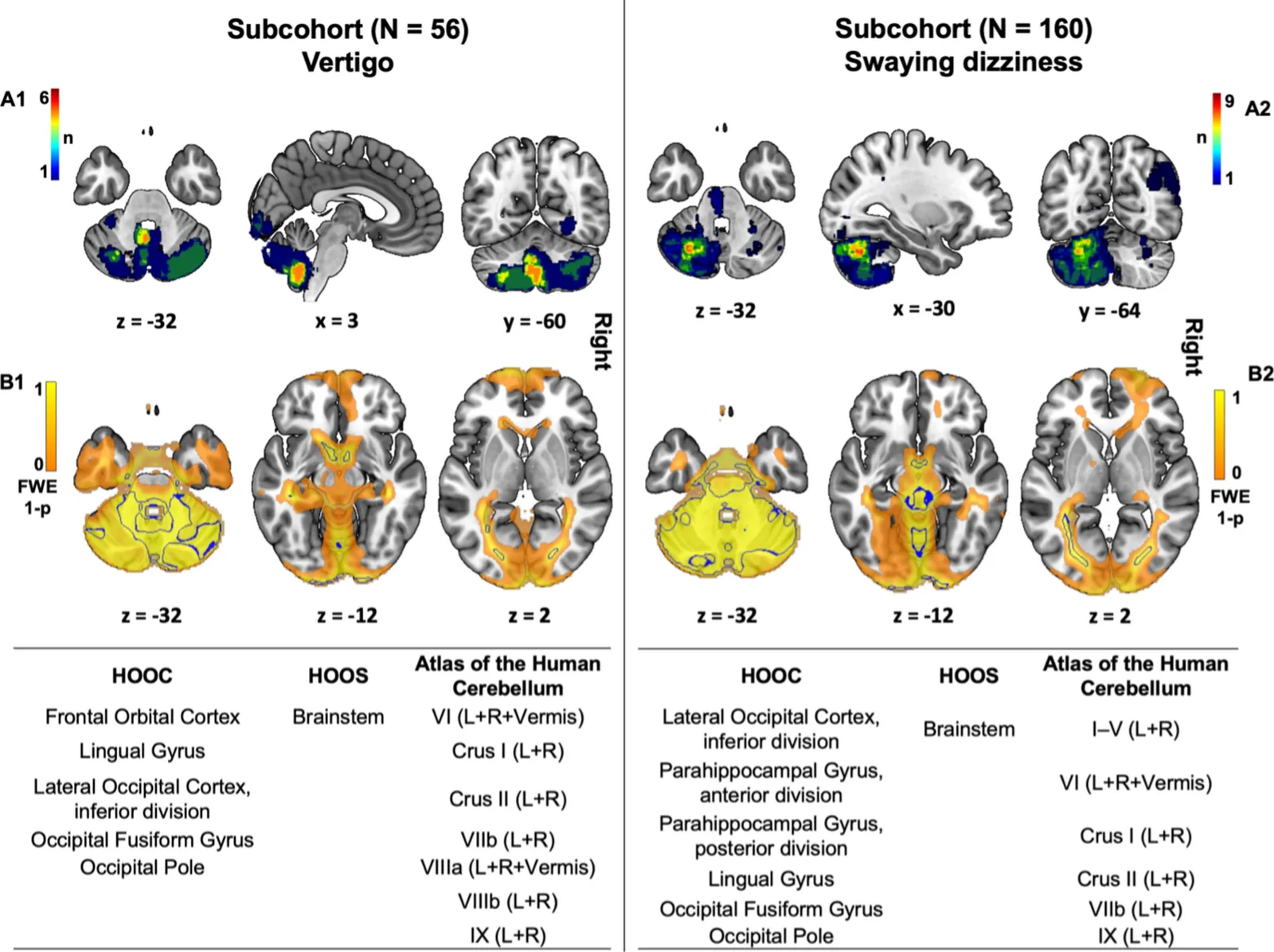
Post hoc exploratory analysis based on retrospective emergency department records. **A1** Lesion heatmap for patients with vertigo (*n* = 14). **B1** FWE-corrected vertigo-related network. **A2** Lesion heatmap for patients with swaying dizziness (*n* = 40). **B2** FWE-corrected swaying dizziness-related network. The tables below represent the atlas query results of significantly connected regions within each network. Solid contours in B1 and B2 delineate regions of significant positive connectivity (FWE 1-p ≥ 0.95)

Out of the 157 patients with dizziness, 14 (8.9%) had vertigo, 40 (25.5%) had swaying dizziness, and 103 (65.6%) had other (non-specific) dizziness. LNM for vertigo (*n* = 56; including 42 age- and sex-matched controls) not only revealed a significant network (pFWE < 0.05), showing the strongest connectivity centered around the vermis of VI and VIIIa, but also included connectivity to the occipital cortex and frontal orbital areas (Fig. 5 B1). On the other hand, the LNM for swaying dizziness (*n* = 160; 40 cases, 120 matched controls) identified a network with strongest connectivity to hemispheric regions including occipital and parahippocampal regions, and a notably broader distribution across the cerebellar hemispheres, encompassing bilateral lobules I–VI, Crus I–II, VIIb, and IX (Fig. 5 B2), when pFWE < 0.05. Although both networks were spatially similar to each other (MSR; Spearman *r* = 0.8, *p* < 0.001) and to the main dizziness network in the entire cohort (MSR, *p* < 0.001 for both; Spearman *r* = 0.70 for the vertigo network and *r* = 0.80 for the swaying dizziness network), they exhibited distinct patterns of cerebellar involvement. See Fig. 5 for lesion distributions and atlas queries.

#### Spatial overlap of LNM networks with vestibular cortex

**Fig. 6:**
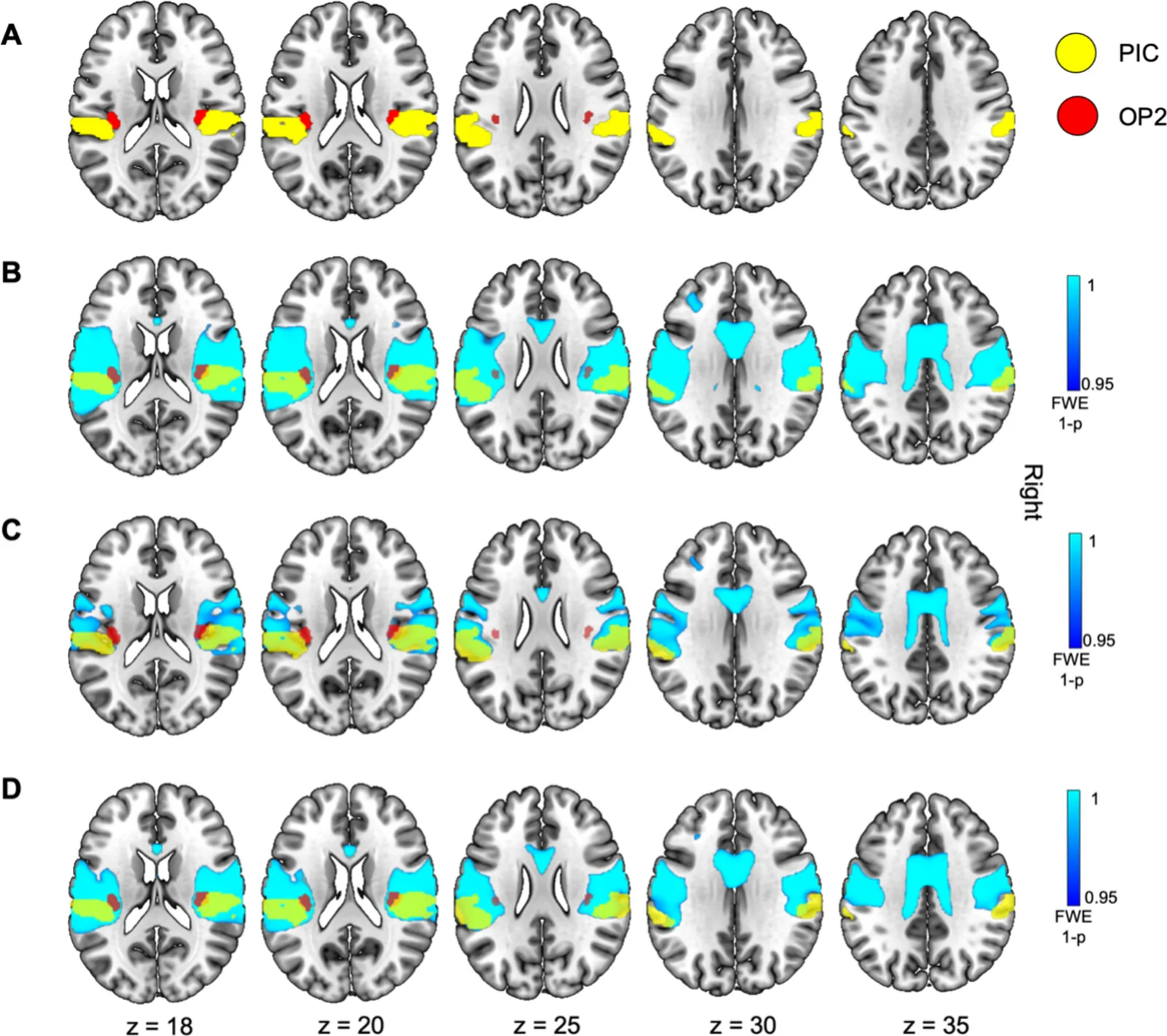
Spatial overlap between negative connectivity networks and the vestibular cortical regions posterior insular cortex (PIC) and OP2. **A** Anatomical locations of PIC (yellow) and area OP2 (red). **B** Unadjusted significant negative connectivity network derived from the entire cohort (*N* = 484) showing overlap with PIC and OP2. **C** Negative connectivity network associated with vertigo (*N* = 56) overlapping with PIC and OP2. **D** Negative connectivity network associated with swaying dizziness (*N* = 160) overlapping with PIC and OP2. The color bar indicates FWE-corrected 1-p values, ranging from dark blue to light blue. All maps were overlaid on slices of the 100-µm MNI152 template

To assess overlap with classical vestibular regions, we extracted regions of interest (ROIs) for OP2 (from the Julich Atlas [48]) and PIC (defined as the rostroventral supramarginal gyrus in the Brainnetome Atlas [11, 49]). Overlaying the dizziness-related networks from the full cohort and both dizziness subtypes revealed consistent spatial overlap with vestibular cortex regions, specifically within the significantly negative (pFWE < 0.05) networks (Fig. 6**)**.

## Discussion

In this study, approximately one-third of patients with acute ischemic stroke reported dizziness at hospital admission. Although dizziness is typically attributed to infratentorial lesions [50], nearly half of the affected patients had exclusively supratentorial strokes. LNM of stroke-related dizziness revealed a common symptom network involving the left cerebellar lobules VI and Crus I–II, with strong connectivity to higher-order visual cortices. This network remained consistent regardless of lesion location (infratentorial vs. supratentorial lesions) and following adjustment for concomitant hemianopia. In exploratory analyses of dizziness subtypes, vertigo was more strongly associated with the cerebellar vermis, while swaying dizziness showed broader connectivity to cerebellar hemispheres.

Patients included in the current analysis were relatively mildly affected (median NIHSS score of 2) in terms of stroke severity, in line with the inclusion criterion of no or non-disabling deficits at the time of inclusion in the INSPiRE-TMS trial [17]. As previously described, patients presenting with dizziness at stroke onset were significantly more often females (Table 1) [4]. This sex difference may reflect etiological variations in dizziness, as females are more prone to vestibular migraine and autoimmune inner ear disorders, which may increase their susceptibility to dizziness [2]. Interestingly, patients with acute dizziness reported greater subjective mobility impairment compared to those without dizziness, despite lower rates of paresis, highlighting the impact of dizziness on perceived functional limitations [51]. In our cohort, 22% of patients with supratentorial stroke (*n* = 77/347) reported dizziness, which is higher than reported previously in the literature [10, 52]. This may reflect the inclusion of milder stroke cases, as patients with severe or hemiplegic strokes may be less likely to report dizziness as a primary symptom.

As expected, dizziness was more frequently associated with VB strokes. Notably, patients who experienced dizziness also had significantly higher rates of concomitant hemianopia. Hemianopia—typically caused by lesions in the PCA territory—results in loss of half the visual field [53]. Although hemianopia is likely to contribute to a subjective sense of dizziness [54], additional critical anatomical regions within the PCA territory, such as the calcarine, occipitotemporal, and occipitoparietal cortices, may also be implicated in the acute sensation of dizziness when damaged, as these areas are involved in both primary and higher-order visual processing as well as spatial orientation [55]. It is important to note that the PCA territory is predominantly supplied by the posterior circulation. Accordingly, subtle ischemic changes in the cerebellum or brainstem may be present in patients with embolic PCA infarcts but remain undetectable on conventional MRI. We cannot exclude the possibility that such undetected lesions contribute to the occurrence of dizziness in these patients. Additionally, because hemianopia and dizziness may share a common PCA territory lesion origin, hemianopia could act as a confounder, a mediator, or a collider in this context, and our data cannot distinguish among these possibilities. The close similarity between covariate-adjusted and unadjusted LNM networks should therefore be read as indicating that our findings are not driven solely by co-occurring hemianopia, rather than as a formal confounding control.

Both voxel-based and atlas-based LSM revealed significant associations between dizziness and cerebellar regions, including the left lobules VI and IX, Crus I–II, VIIb, VIIIa, Vermis IX, and right lobule IX (Fig. 3). These areas, located in the posterior cerebellum, are implicated in visuospatial processing, oculomotor control, and multisensory integration [56, 57]. Significant LSM clusters also showed positive connectivity with visual regions such as the superior and inferior divisions of lateral occipital cortex, occipital pole, occipital fusiform gyrus, and lingual gyrus––key areas for primary and higher-order visual processing [58–60] . Together, these findings support an important role of the cerebellum in broadly defined post-stroke dizziness and highlight its connectivity with higher-order visual networks.

LNM of dizziness across the entire patient cohort, independent of lesion location (infra- or supratentorial), revealed a significant, bilateral network encompassing the cerebellum, brainstem, occipital pole, occipital fusiform gyrus, and lingual gyrus (Fig. 4A). The cerebral regions implicated are primarily involved in visual processing: the occipital pole includes primary visual cortex (V1), while the lingual gyrus and fusiform gyrus (V2) are involved in higher-order functions such as object recognition and spatial orientation [58–60]. The cerebellar regions identified (Supplementary Table S2) support vestibular control (Lobule V), visuospatial processing (Lobules VI, VIIb, Crus I–II), self-motion prediction and orientation (Lobule IX), and sensorimotor integration (Lobule VIIIa) [56, 57, 61].

Together, these findings are compatible with the disruption of distributed multisensory (visual–vestibular–proprioceptive) processing networks involved in spatial orientation and balance. Sensitivity analyses—including covariate-adjusted and alternative MRI preprocessing pipelines—showed high spatial similarity with the primary dizziness network, supporting robustness and consistency of the observed network pattern (Supplementary Fig. S1).

To further explore cortical contributions to stroke-induced dizziness, we stratified LNM by lesion location (infratentorial vs. supratentorial). Both networks (Fig. 4) showed significant positive connectivity primarily to the left cerebellar hemisphere and occipital regions, partial spatial overlap with each other, and statistically significant similarity to the full-cohort dizziness network. The supratentorial network additionally involved the occipital fusiform gyrus, lingual gyrus, and lateral occipital cortex (LOCi; Supplementary Table S2). The LOCi is involved in object recognition, shape processing, and spatial orientation via visual cue integration [55, 62]. Notably, left-lateralized cerebellar connectivity—observed in the supratentorial subgroup—may reflect visuospatial integration through contralateral projections to the right parietal cortex, a key vestibular hub [63–65]. These visual networks remained spatially similar after adjusting for covariates (Supplementary Fig. S1C).

Importantly, these supratentorial findings emerge from LNM based on a normative healthy-subject connectome and, as discussed in our Limitations, indicate potential network relationships rather than patient-specific functional disruption; they should therefore be interpreted more cautiously than the direct lesion localization obtained from LSM. This caution applies in particular to the occipital connectivity: because hemianopia was substantially more frequent among dizzy patients (35% vs. 16%) and may share a common PCA territory lesion origin with dizziness, it could act as a confounder, mediator, or collider, and our data cannot distinguish among these possibilities. The close similarity between covariate-adjusted and unadjusted networks should be read as indicating that our findings are not driven solely by co-occurring hemianopia, rather than as a formal confounding control.

In a post hoc exploratory analysis, LNM was performed separately for patients with vertigo and swaying dizziness (Fig. 5). Both networks showed high spatial similarity (Spearman r = 0.80, *p* < 0.001), particularly in occipital regions, yet distinct patterns emerged: vertigo was more strongly associated with medial cerebellar connectivity, notably the posterior vermis (lobules VI and VIIIa), whereas swaying dizziness involved broader cerebellar hemispheric connectivity. The vermis, part of the limbic cerebellum, has been linked to visuospatial processing, potentially relating to the rotational features of vertigo [64, 65]. These preliminary differences are hypothesis generating and require validation in larger, prospectively phenotyped samples, given the limitations of retrospective symptom classification, the non-specific terminology commonly used in the acute stroke setting, and the small number of patients with vertigo (*n* = 14), and the exclusion of patients with non-specific dizziness from the subtype analyses.

The dizziness network identified in our study differs notably from the previously published “cortical vertigo network,” [13] which was derived from 23 case reports of isolated cortical vertigo. That network included bilateral regions such as the parieto-insular cortex (PIC), area OP2, somatosensory and visual areas, thalamus, and cerebellar lobule VIII––closely aligning with the established vestibular cortex involved in multisensory integration. In contrast, our dizziness-related network consistently showed *positive* connectivity to occipital (visual) areas and *negative* connectivity to core vestibular regions, including PIC and OP2 (Fig. 6), a pattern that was remarkably consistent across all subgroup analyses. This divergence may reflect the limitations of normative connectome-based LNM in capturing patient-specific cortical network organization, particularly given the ongoing uncertainty surrounding the interpretation of negative functional connectivity in LNM [16] and resting-state functional connectivity in general [68]. Alternatively, it may reflect the reciprocal inhibitory interaction between visual and vestibular systems, whereby increased visual engagement is accompanied by relative suppression of vestibular cortical activity during postural control and spatial orientation [69]. However, our data cannot distinguish between these explanations, and both remain to be validated in future studies using individualized functional imaging. Accordingly, our findings should be interpreted as identifying a distributed network associated with stroke-related dizziness that encompasses visual, cerebellar, and vestibular systems, rather than as direct evidence of impaired vestibular cortical processing or visual–vestibular integration per se.

### Strengths and limitations

A major strength of this study is the use of a well-characterized cohort of patients with minor ischemic stroke (modified Rankin Scale ≤ 2) derived from a multicenter randomized controlled trial. Furthermore**,** data on stroke-related dizziness are often lacking in larger cohorts, as this symptom is not captured by standard severity scales such as the NIHSS. A key strength of this study is that new dizziness associated with the acute cerebrovascular event was prospectively assessed by trained personnel upon admission to the stroke unit.

However, this study has several important limitations that warrant discussion. First, although dizziness was prospectively assessed via questionnaire as part of the study protocol, it remains a patient-reported outcome inherently susceptible to misclassification. Despite specifically inquiring about new dizziness related to the acute cerebrovascular event, we cannot fully exclude that some patients may have reported pre-existing or chronic symptoms due to misinterpretation. Additionally, while symptom assessment focused on the acute clinical presentation, we were unable to systematically differentiate between spontaneously reported and elicited symptoms [70]. A major limitation of this study is the absence of systematic oto-neurological examination, including characterization of nystagmus, ataxia, and other vestibular signs. Consequently, dizziness could only be classified according to patient-reported symptoms and routine clinical documentation, precluding objective differentiation of vestibular syndromes. Future prospective studies incorporating standardized oto-neurological assessments are warranted. Therefore, the identified network may reflect a broader syndrome of post-stroke spatial-sensory disturbance rather than a vestibular-specific network. We acknowledge that prospective collection of structured oto-neurologic data will be an important direction for future work. Second, the study population was derived from the INSPiRE-TMS trial, which enrolled patients with non-disabling strokes, thereby limiting representation of more severe strokes—particularly PCA infarcts in which dizziness may be a more prominent and diagnostically critical feature—and consequently restricting the generalizability of our findings. Third, only patients with DWI-positive lesions were included; however, small or early posterior circulation strokes may be DWI negative, potentially leading to systematic exclusion of a clinically relevant subgroup [71]. Fourth, the post hoc exploratory analysis of dizziness subtypes (vertigo, swaying dizziness, and other) was based on clinical documentation rather than standardized assessments, and is therefore subject to imprecision and variability in symptom reporting [72].

A methodological constraint of the LSM analysis concerns the voxel inclusion threshold. Because LSM requires a minimum number of overlapping lesions at each voxel, only 2.2% of total brain voxels were eligible for analysis, concentrated in cerebellar and other infratentorial structures, with sparser coverage of supratentorial regions, so that large portions of the supratentorial cortex were effectively untested. The absence of a widespread significant supratentorial LSM signal should therefore not be read as evidence against symptom-relevant supratentorial localization. Consistent with this, applying LNM to the significant LSM clusters revealed positive connectivity to occipital cortical regions, in line with our other findings.

Furthermore, LNM is based on a normative connectome from healthy individuals 37 and does not account for potential stroke-specific alterations or connectome disruptions due to previous vascular pathology [73]. In addition, arterial territory assignment was performed using a digital atlas, 28 which may have introduced misclassification, especially for lesions near vascular border zones or in anatomically variable regions such as the PCA territory [74]. However, this method likely offers greater reproducibility compared to manual classification. Finally, the use of different scanning protocols across 1.5-Tesla and 3-Tesla scanners introduces variability and a potential bias in terms of image acquisition/quality and subsequent preprocessing pipelines. Although no MRI preprocessing pipeline is entirely error free, the consistent results obtained using an alternative pipeline in our sensitivity analyses support the robustness of our findings. Furthermore, neurological assessments were conducted across multiple INSPiRE-TMS centers, which inherently introduces heterogeneity in clinical evaluations despite the use of standardized protocols.

Recent methodological work has highlighted important constraints of LNM [75], demonstrating that LNM outcomes are shaped by fundamental properties of resting-state functional connectivity. We acknowledge that rs-fMRI networks are low-dimensional and dominated by a limited set of large-scale components. Accordingly, the spatial similarity observed across the LNM analyses in the present study should not be interpreted as exclusively reflecting symptom-specific neurobiology, but may also partly arise from this shared low-dimensional network architecture. To ensure robust inference on symptom-specific connectivity patterns and control for false positives, we employed permutation-based testing with 5,000 permutations and family-wise error correction, consistent with recently proposed practices for LNM statistical inference [76]. In addition, statistical significance of spatial similarity was assessed using Moran's spectral randomization, which accounts for spatial autocorrelation but does not fully isolate symptom-specific connectivity from the intrinsic low-dimensional organization of the normative connectome. Therefore, we interpret the global similarity across LNM networks with caution and place greater emphasis on the localized convergence and peak connectivity patterns observed across analyses stratified by lesion location and dizziness subtype. Specifically, we identified a common symptom-related network involving left cerebellar lobules VI and Crus I–II with strong connectivity to higher-order visual cortices. Supratentorial dizziness-related networks showed prominent occipital involvement, whereas infratentorial analyses revealed dissociable patterns. Similarly, exploratory analyses of dizziness subtypes suggested preferential involvement of the cerebellar vermis in vertigo and broader cerebellar hemispheric connectivity in swaying dizziness.

## Conclusion

We identified a statistically significant lesion network associated with broadly defined post-stroke dizziness involving cerebellar and occipital regions. This network remained consistent across lesion locations and exploratory symptom subtypes, suggesting the involvement of distributed visual, vestibular, and multisensory brain systems. These findings provide network-level correlates of post-stroke dizziness and offer a framework for future studies incorporating detailed vestibular phenotyping and individualized functional imaging. Improved characterization of these network correlates may contribute to better recognition of post-stroke dizziness and inform future diagnostic and therapeutic strategies.

## Supplementary Information

Below is the link to the electronic supplementary material.

Supplementary file1 (DOCX 784 KB)

## Data Availability

Data supporting the results of this study can be requested by qualified researchers who provide a methodologically reasonable proposal by contacting the corresponding author. The request will be thoroughly examined, and data will be provided in compliance with data protection and privacy laws upon a signed data access agreement.

## Abbreviations

ANTsPy: Advanced Normalization Tools in Python
ACA: Anterior cerebral artery
BET: Brain Extraction Tool
DWI: Diffusion-weighted imaging
ED: Emergency department
FLAIR: Fluid-attenuated inversion recovery
FSL: FMRIB Software Library
FWE: Family-wise error
GLM: General linear model
INSPiRE-TMS: Intensified Secondary Prevention intending a Reduction of vascular re-events after TIA or Minor Stroke
LSM: Lesion symptom mapping
LNM: Lesion network mapping
LOCi: Inferior division of lateral occipital cortex
MNI: Montreal Neurological Institute
MCA: Middle cerebral artery
MSR: Moran’s spectral randomization
NIHSS: National Institutes of Health Stroke Scale
OP2: Operculum area 2
PCA: Posterior cerebral artery
TOAST: Trial of Org 10,172 in Acute Stroke Treatment
TFCE: Threshold-free cluster enhancement
VB: Vertebrobasilar

## Code availability

Open-source software were used for the preprocessing and analysis of the data: Lead-DBS (https://github.com/netstim/leaddbs); NiiStat Software (https://www.nitrc.org/projects/niistat); FSL version 6.0.6.4 (https://fsl.fmrib.ox.ac.uk/fsl/fslwiki/); ANTsPy (https://github.com/ANTsX/ANTsPy); BrainSpace toolbox (https://brainspace.readthedocs.io/en/latest/); R studio (https://posit.co/download/rstudio-desktop/).
