# Supplementary material for: Neuroanatomical correlates of stroke-related dizziness and vertigo: secondary analysis from the INSPiRE-TMS trial
Source: J Neurol. 2026 Aug 12;273(9):524. doi: 10.1007/s00415-026-14033-3 (PMC13469211; doi:10.1007/s00415-026-14033-3)
Supplement: Supplementary file 1 [file 415_2026_14033_MOESM1_ESM.docx]

-**Supplementary Material-**

**Neuroanatomical Correlates of Stroke-Related Dizziness and Vertigo: Secondary Analysis from the INSPiRE-TMS Trial**

Yunyou Tang, Uchralt Temuulen, Ana Sofia Rios, Ahmed Khalil, Michael Ahmadi, Andreas Horn, Matthias Endres, Heinrich J Audebert, Anna Kufner

Supplementary Table 1. Comparison of two MRI preprocessing pipelines (P2)

Supplementary Figure 1. Sensitivity analysis (P3)

Supplementary Table 2. Structures connected to dizziness networks in permutation analysis of LNM (P4)

Supplementary Table 3. The functionally connected regions of the adjusted *Supratentorial dizziness-related network* (P5)

|  | **Original MRI preprocessing pipeline** | **Alternative MRI preprocessing pipeline** |
| --- | --- | --- |
| **Basis** | Perform ANTsPy image registration with optional ROI transformation. | Perform two-stage ANTsPy registration with optional ROI handling, quality checks, and fallbacks. |
| **Workflow** | **Rigid Registration** - Aligns moving image using rotation and translation.  **Non-linear Registration (SyN)** - Refines alignment with diffeomorphic warping.  **ROI Processing**  - Applies identical transforms to ROI mask using nearest-neighbor interpolation. | **Linear Registration (Affine, including Rigid)**  - Applies rotation, translation, scaling, and shearing.  **Non-linear Registration (SyN)**  - Refines alignment via diffeomorphic deformation.  **ROI Processing**   - Transforms ROI mask through the same stages with nearest-neighbor interpolation.  **Fallback Strategies for ROI**  - Retries failed steps with alternative metrics (meansquares).  **File Handling**  - Skips existing outputs unless force=True; reuses transforms if available. |
| **The number of final cohorts** | N = 484 | N = 414 |

**Supplementary Table 1.** Comparison between the original and alternative MRI preprocessing pipelines implemented in ANTsPy. The table outlines differences in registration stages, ROI handling, and fallback strategies. ROI: Region of Interest; SyN: Symmetric Normalization

**Supplementary Figure 1.** Sensitivity analysis. ***A)*** Adjusted dizziness network derived from permutation analysis of the presence of dizziness (yes/no) at the acute stage post stroke in the total cohort (N = 461) after adjusting for sex, age, lesion volume, and the presence of hemianopsia (binary, yes/no). ***B)*** Unadjusted dizziness network using lesions processed via an alternative MRI preprocessing pipeline (N = 414). ***C)*** Adjusted *supratentorial dizziness-related network* at the acute stage post stroke in the total cohort (N = 331) after adjusting for sex, age, lesion volume, and the presence of hemianopsia (binary, yes/no). The maps were overlaid on slices of a 100µm brain scan in MNI152 space, with color bars representing 1 - p FWE-corrected voxel values.

| **Map / Atlas** | **HOOC** | **HOOS** | **Atlas of the Human Cerebellum** |
| --- | --- | --- | --- |
| **Unadjusted dizziness-related network irrespective of lesion location**  **(N = 484)** | Lingual Gyrus | Brainstem | Left V |
|  | Occipital Fusiform Gyrus |  | Right V |
|  | Occipital Pole |  | Left VI |
|  |  |  | Vermis VI |
|  |  |  | Right VI |
|  |  |  | Left Crus I |
|  |  |  | Right Crus I |
|  |  |  | Left Crus II |
|  |  |  | Right Crus II |
|  |  |  | Left VIIb |
|  |  |  | Right VIIb |
|  |  |  | Right VIIIa |
|  |  |  | Left IX |
|  |  |  | Right IX |
| ***Infratentorial dizziness-related network***  **(n = 137)** |  |  | Left VI |
|  |  |  | Vermis VI |
|  |  |  | Right VI |
|  |  |  | Right Crus I |
|  |  |  | Left Crus II |
|  |  |  | Vermis Crus II |
|  |  |  | Right Crus II |
|  |  |  | Left VIIb |
|  |  |  | Vermis VIIb |
|  |  |  | Right VIIb |
|  |  |  | Left VIIIa |
|  |  |  | Vermis VIIIa |
|  |  |  | Right VIIIa |
|  |  |  | Left VIIIb |
|  |  |  | Vermis VIIIb |
|  |  |  | Right VIIIb |
|  |  |  | Left IX |
|  |  |  | Vermis IX |
|  |  |  | Right IX |
|  |  |  | Left X |
|  |  |  | Right X |
| ***Supratentorial dizziness-related network***  **(n = 347)** | Lateral Occipital Cortex, inferior division (LOCi) |  | Left VI |
|  | Lingual Gyrus |  | Left Crus I |
|  | Occipital Fusiform Gyrus |  | Left Crus II |
|  | Occipital Pole |  |  |

**Supplementary Table 2.** Structures connected to dizziness networks at the acute stage post stroke in permutation analysis of LNM. HOOC: Harvard Oxford Cortical Structural Atlas, HOOS: Harvard Oxford Subcortical Structural Atlas, and FNIRT-normalized Cerebellar Atlas in MNI152 space.

| **Map / Atlas** | **HOOC** | **HOOS** | **Atlas of the Human Cerebellum** |
| --- | --- | --- | --- |
| **Adjusted dizziness network irrespective of lesion location  (N = 461)** | Occipital Pole | Brain-Stem | Left I-IV |
|  |  |  | Right I-IV |
|  |  |  | Left V |
|  |  |  | Right V |
|  |  |  | Left VI |
|  |  |  | Vermis VI |
|  |  |  | Right VI |
|  |  |  | Left Crus I |
|  |  |  | Right Crus I |
|  |  |  | Left Crus II |
|  |  |  | Right Crus II |
|  |  |  | Left VIIb |
|  |  |  | Right VIIb |
|  |  |  | Left VIIIa |
|  |  |  | Right VIIIa |
|  |  |  | Left VIIIb |
|  |  |  | Right VIIIb |
|  |  |  | Left IX |
|  |  |  | Right IX |

**Supplementary Table 3.** Structures connected to the adjusted dizziness network irrespective of lesion location (N = 461) by controlling for sex, age, lesion volume and the presence of hemianopsia. HOOC: Harvard Oxford Cortical Structural Atlas, HOOS: Harvard Oxford Subcortical Structural Atlas, and FNIRT-normalized Cerebellar Atlas in MNI152 space.
